# The Missouri Dental College

**Published:** 1868-04

**Authors:** 


*The Missouri Dental College.*

The second annual commencement of this College was held on the 26th of February, at O'Fallon Hall. The degree of Doctor of Dental Surgery was conferred upon the following three gentlemen constituting the graduating class:—John R. Mathews, Alfred C. Sloan and William L. Thomas. A respectable audience, composed of the numerous friends of the Institution, filled the Hall. A very excellent parting address to his fellow-students was given by Mr. John R. Mathews. The valedictory address was delivered by Prof. H. E. Peebles. It was full of earnest advice to the students, and gave encouragement to the friends of the College as to its future.

The number of students in attendance upon the lectures the past session was ten.

